# Continued decline in the incidence of myocardial infarction beyond the COVID-19 pandemic: a nationwide study of the Swedish population aged 60 and older during 2015–2022

**DOI:** 10.1007/s10654-024-01118-4

**Published:** 2024-04-23

**Authors:** Anna C. Meyer, Marcus Ebeling, Enrique Acosta, Karin Modig

**Affiliations:** 1 Unit of Epidemiology, Institute of Environmental Medicine, Karolinska Institutet, PO Box 210, 17177 Stockholm, Sweden; 2 Centre for Demographic Studies (CED), Carrer de Ca N’Altayó, Edifici E2 Universitat Autònoma de Barcelona, Bellaterra, 08193 Bellaterra, Spain; 3 Max Planck Institute for Demographic Research, Konrad-Zuse-Str. 1, 18057 Rostock, Germany

**Keywords:** Myocardial infarction, COVID-19, Epidemiological monitoring, Public health

## Abstract

The number of myocardial infarctions declined during the early COVID-19 pandemic but mechanisms behind these declines are poorly understood. COVID-19 infection is also associated with an increased risk of myocardial infarction which could lead to higher incidence rates in the population. This study aims to shed light on the seemingly paradoxical relationship between COVID-19 and myocardial infarction occurrence on the population level by exploring long-term trends in incidence rates, case fatality, and proportion of patients dying before reaching a hospital. Our work is based on a linkage of administrative registers covering the entire population aged 60 + in Sweden. Considering both long-term trends since 2015 and seasonal variability, we compared observed incidence, case fatality, and proportions of patients hospitalized to expected values during 2020–2022. Despite more than 200 laboratory-confirmed COVID-19 cases per 1000 inhabitants by the end of 2022, incidence rates of myocardial infarction continued to decline, thus following the long-term trend observed already before 2020. During the first pandemic wave there was an additional incidence decline corresponding to 13% fewer myocardial infarctions than expected. This decline was neither accompanied by increasing case fatality nor by lower shares of patients being hospitalized. We found no increase in the population-level incidence of myocardial infarction despite large-scale exposure to COVID-19, which suggests that the effect of COVID-19 on myocardial infarction risk is not substantial. Increased pressure on the Swedish health care system has not led to increased risks or poorer outcomes for patients presenting with acute myocardial infarction.

**Supplementary Information:**

The online version contains supplementary material available at 10.1007/s10654-024-01118-4.

## Introduction

During the early phase of the COVID-19 pandemic, the number of hospital admissions for myocardial infarction declined across the world [1–18]. Despite the vast number of published studies, it is still unknown whether the observed declines reflect a real decrease in the risk of myocardial infarction or merely the fact that fewer patients reached a hospital. Several studies hypothesized that reduced care seeking among individuals who experienced symptoms of myocardial infarction led to lower numbers of patients presenting in hospitals [15–18]. Evidence for this hypothesis is, however, mixed. Although delays in the care of myocardial infarction were observed in some settings, several European studies did not find significantly increased delays between symptom onset and first medical contact for patients with myocardial infarction [16, 19, 20]. Moreover, since timely treatment of myocardial infarction is essential, delays in care seeking would likely result in increasing case fatality and in growing proportions of patients dying before reaching the hospital. Lower admission rates for cardiovascular diseases were indeed accompanied by increased case fatality during the first pandemic year in the United States [18] but not in several European countries [4, 8, 14, 21]. However, there are also other hypotheses that could explain the declining number of myocardial infarctions during the early pandemic, such as changes in lifestyle factors, stress levels, and environmental exposures [22]. These hypotheses would result not only in a decline in the number of patients presenting at hospitals but also in an overall decline in incidence rates.

In parallel, COVID-19 infection has been linked to an increase in the risk of cardiovascular diseases, including acute myocardial infarction [23]. This suggests that the initial decline in hospital admissions might eventually turn into increasing incidence rates of myocardial infarction in the long run—especially since COVID-19 affected a large share of the population. Moreover, the cardiovascular damage and exacerbation of cardiovascular disease during infection with the SARS-CoV-2 virus [23, 24] might have affected survival rates for patients with myocardial infarction. Nevertheless, to our knowledge, data reflecting case-fatality throughout the pandemic years have not been presented.

Incidence rates –as well as case fatality– declined continuously during the past decades. Most previous studies did not take this trend into account and directly compared data observed in 2020 to an earlier reference period, usually 2019 [3, 4, 6, 7, 9–11, 13, 16]. In addition, only a few studies have estimated incidence rates based on accurate measures of person-time at risk, considering the increased mortality due to COVID-19 itself. Previous studies were, moreover, often limited to clinical populations [3, 4]. In contrast to administrative registers, the coverage of clinical databases declined during the pandemic [25, 26].

With this study, we explore the seemingly paradoxical scenario in which the number of myocardial infarctions decreased in the early pandemic, but subsequent research revealed an elevated risk of myocardial infarction associated with COVID-19 infection. One could thus hypothesize that the incidence of myocardial infarction increased during the later phases of the pandemic. Here, we present population-wide trends in age-specific incidence rates, case fatality and the proportion of patients hospitalized between the onset of the COVID-19 pandemic and the end of 2022 in Sweden.

## Methods

### Data and study population

This study is based on a linkage of administrative population registers using the unique personal identification number assigned to each Swedish resident. The entire population over the age of 60 residing in Sweden between 2015 and 2022 was identified in the Total Population Register (TPR). Individuals entered the study population in the month of their 60th birthday and were followed until death, emigration, loss to the registers (i.e., no registration in the TPR without recorded death or emigration), or the end of 2022, whichever came first. Based on weekly data on confirmed cases and COVID-19 deaths in Sweden reported by WHO, we defined the first, second, and third pandemic waves as the time periods 23–03-2020 to 12-07-2020, 19-10-2020 to 23-05-2021, and 20-12-2021 to 15-05-2022, respectively [27].

Myocardial infarctions were identified in the Cause of Death Register (CDR) and in the National Patient Register (NPR) using the 10th version of the International Classification of Diseases (ICD) codes. The CDR records death dates of all individuals registered in Sweden together with ICD codes for the underlying and contributing causes of death. The NPR contains all hospital admissions and specialized outpatient care visits in the country together with ICD diagnoses assigned by physicians.

In accordance with the Swedish National Board of Health and Welfare, incident events were defined through ICD-codes I21 or I22 as main or contributing cause of hospitalization or death occurring at least 28 days apart [28]. A comparison with clinical data during 2021 showed that this definition yields a sensitivity of 94% for detecting incident myocardial infarction in Sweden [28]. Older validation studies have further demonstrated excellent positive predictive values (98 and 100%, respectively) [28, 29]. Case fatality was defined as the proportion of individuals dying within 30 days after the occurrence of a myocardial infarction. The proportion of patients reaching the hospital was calculated as the number of incident events identified in the NPR divided by all incident events.

Information on place of residence was available on a yearly basis. For the stratification by geographical region, we therefore distinguished between individuals registered in Stockholm County and those registered elsewhere on December 31st of the previous year. A person contributed person-time at risk as well as disease events to the population of Stockholm County if they were registered there at the end of the previous year.

### Statistical analyses

For each month between January 2015 and December 2022, we calculated person-years at risk by counting the number of days spent at risk of MI for every individual and transforming the total number of days into years. Incidence rates were calculated as the number of incident myocardial infarctions observed divided by person-time at risk for each month. To compare incidence rates during the pandemic to an appropriate reference, i.e., to the expected incidence in absence of the pandemic, we estimated expected monthly myocardial infarctions for the time period March 2020 to December 2022 considering both long-term incidence trends and within-year seasonal variability between January 2015 and February 2020. A quasi-Poisson generalized additive model, including a log-linear component for the long-term secular trend, a cyclic p-spline for seasonality, and an offset component to control for changes in the population at risk, was separately fitted to each age group and sex. Based on these models, we predicted the expected monthly myocardial infarctions from March 2020 to December 2022 in the absence of the pandemic and computed 95% prediction intervals using bootstrapping with 2000 iterations. All analyses were stratified by sex and reported separately for four age groups (60–69, 70–79, 80–89 and 90 or older).

### Sensitivity analyses

In sensitivity analyses, we extracted additional data on outpatient care (e.g., visits to outpatient emergency centers) from the NPR. We calculated the number of incident events based on data from inpatient, outpatient, and death records and calculated the proportions of incident events identified in each data source during the first period of the pandemic (March to December 2020). A marked decline in outpatient care utilization could indicate reduced care seeking by patients. Second, we calculated the number of events that could only be identified through outpatient diagnoses, i.e., that had no matching record in either inpatient or death records, which could indicate limited sensitivity of identifying myocardial infarction in the latter two sources. Note that for outpatient records, we only included those with a main diagnosis of myocardial infarction and excluded records with a code for follow-up examinations (ICD-10: Z09).

## Results

**Fig. 1:**
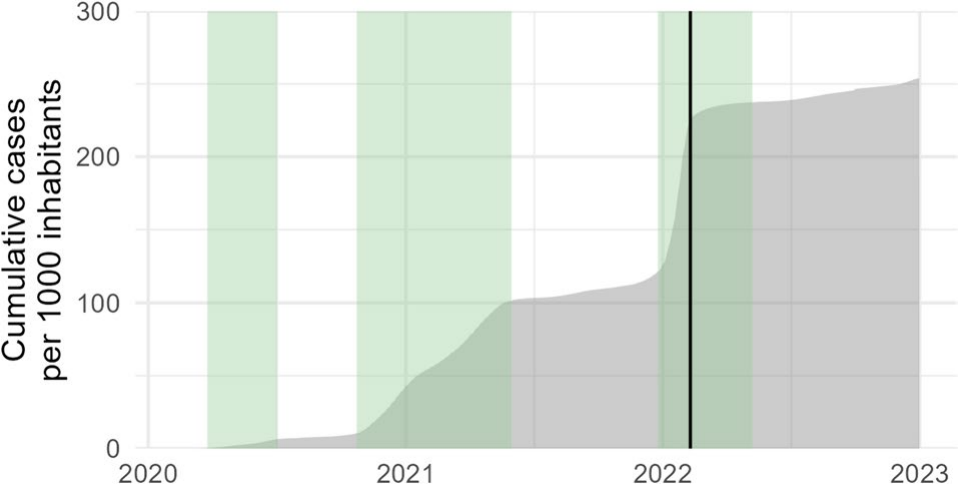
Cumulative number of confirmed COVID-19 cases per 1000 inhabitants in Sweden (shaded grey area) [24]. The black vertical line indicates the date on which most testing for COVID-19 was stopped by the Swedish government (09–02-2022). Vertical green bands show the first three pandemic waves

The first three waves of the COVID-19 pandemic in Sweden (green) together with the cumulative number of confirmed COVID-19 cases per 1000 inhabitants (gray) are shown in Fig. 1. Since testing capacities were limited in the early pandemic and recommendations to test all suspected cases in laboratories were effectively stopped by the Swedish government during February 2022, the cumulative proportion of the Swedish population affected by the virus likely exceeds the numbers shown in Fig. 1.

### Time trends in incidence rates of myocardial infarction

**Fig. 2:**
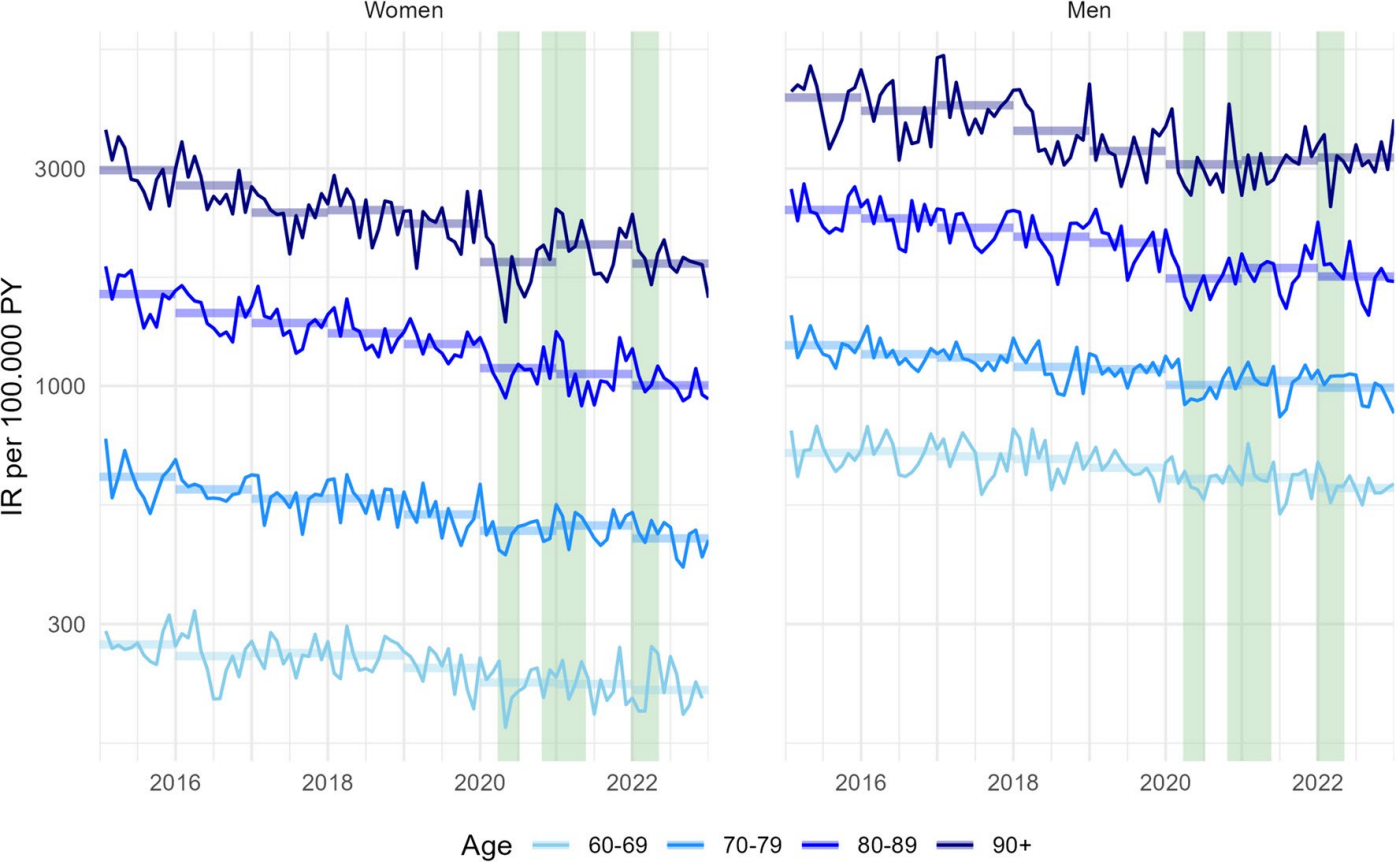
Monthly and annual incidence rates of myocardial infarction per 100,000 person-years in the Swedish population aged 60 and older stratified by sex and age group, January 2015 to December 2022. Annual incidence rates are shown as thick horizontal lines (transparent). Thin lines reflect monthly incidence rates. Vertical green bands indicate three pandemic wave periods in Sweden

Figure 2 shows trends in annual and monthly incidence rates of myocardial infarction between January 2015 and December 2022 stratified by sex and age group. Annual incidence rates (shown as horizontal lines) declined consistently already before 2020. Between 2015 and 2022, declines in annual incidence rates ranged from 16.2% (men aged 60–69) to 37.7% (women aged 90 and older). Declines in annual rates were roughly linear from 2015 through 2022 with the exception of 2020, which deviated from overall trends by exhibiting lower incidence rates. Monthly incidence rates followed a seasonal pattern with a tendency toward lower rates during summer months and higher rates in December and January (Fig. 2).

**Fig. 3:**
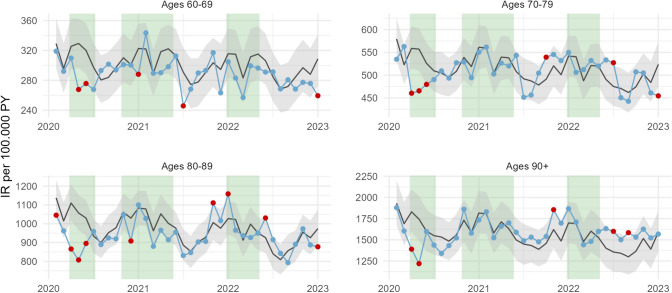
Expected (black lines) and observed (blue lines) incidence rates of myocardial infarction per 100,000 person-years in the Swedish population over the age of 60 from March 2020 to December 2022. Expected incidence rates are based on trends since 2015 and shown together with 95% prediction intervals (gray shading). Highlighted dots indicate observed incidence rates that fell outside the prediction intervals. Vertical bands indicate three pandemic wave periods in Sweden

Figure 3 shows the expected (black line) and observed (blue line) incidence rates of myocardial infarction together with 95% prediction intervals from March 2020 to December 2022 in four age groups. During the first wave of the pandemic, incidence rates were consistently lower than expected for all age groups, although the lower level did not fall outside the prediction interval during all months. In contrast, no consistent deviations from expected numbers were observed during the pandemic’s second and third waves or during the remaining months in 2020 to 2022. The pattern of lower-than-expected incidence rates during the first pandemic wave but no consistent deviations from expected rates thereafter was consistent among men and women (Supplementary Fig. 1) and in Stockholm County as well as the rest of Sweden (Supplementary Fig. 2).

The total number of myocardial infarctions during March to June 2020, i.e., the first pandemic wave (n = 6095), was considerably lower than that during the same period in 2019 (n = 7126), corresponding to a decline of 14.5%. When considering long-term trends, seasonality, and the changing population composition, we estimated approximately 900 (13.0%) fewer myocardial infarctions than expected, largely clustered in age groups between 70 and 89 during March and April 2020 (Fig. 2).

### Case fatality and proportion of patients receiving hospital care

**Fig. 4:**
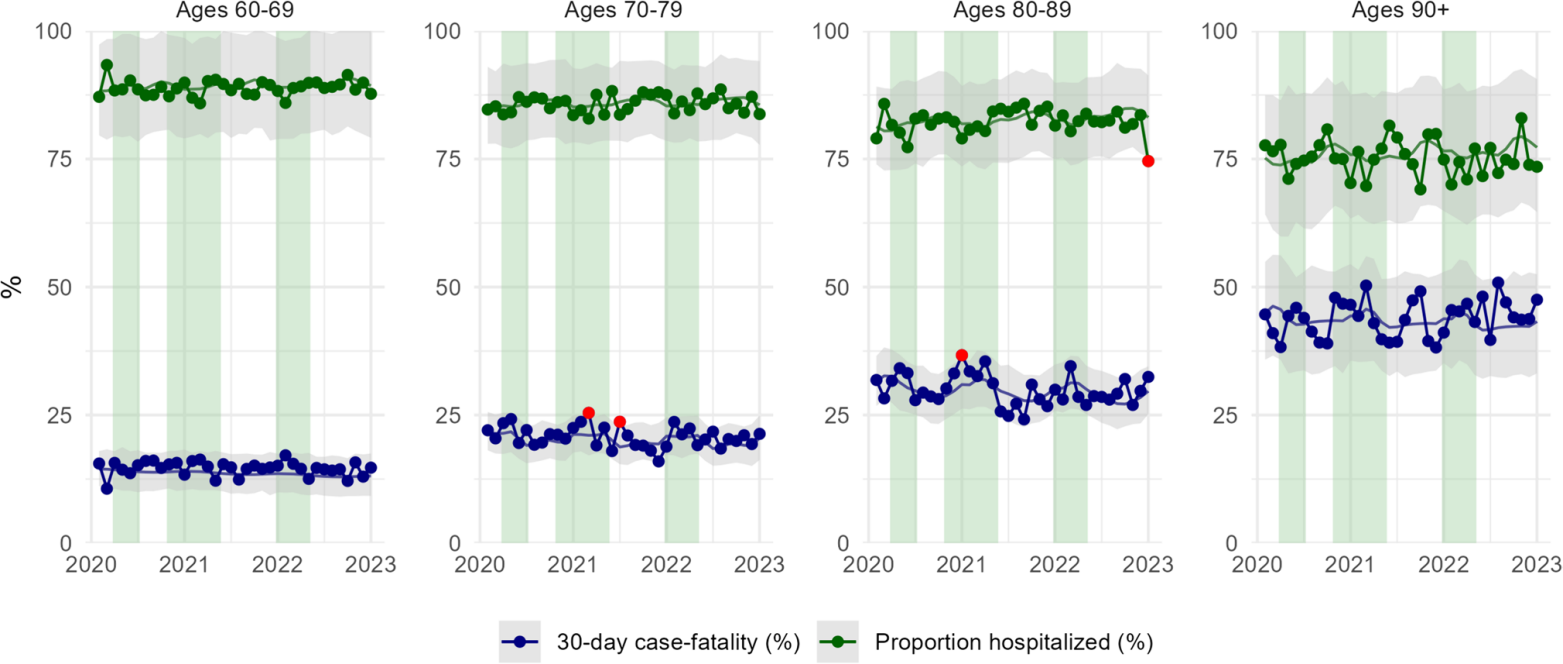
Proportion of myocardial infarction cases dying within 30 days (case fatality, lower graphs) and proportion of individuals with myocardial infarction receiving hospital care (upper graphs) in the Swedish population aged 60 and older in four age groups, March 2020 to December 2022. Shaded gray areas show 95% prediction intervals. Highlighted dots indicate observed incidence rates that fell outside the prediction intervals. Vertical green bands indicate three pandemic wave periods in Sweden

Figure 4 shows proportions of individuals with incident myocardial infarction who died within 30 days as well as proportions of individuals receiving hospital care between March and December 2022 together with 95% prediction intervals. The respective data stratified by sex are shown in Supplementary Fig. 3. From March to June 2020, 25.5% of all patients died within 30 days of experiencing a myocardial infarction compared with 24.9% during the same period in 2019. Case fatality observed in individual months during 2020–2022 was neither consistently higher nor lower than expected proportions.

The average proportion of incident myocardial infarctions receiving hospital care between March and June overall increased from 81.0% in 2015 to 83.7% in 2019 and remained at 83.6% in 2020. From March to June 2022, 84.6% received hospital care. There were no consistent deviations from expected values (Fig. 4). We observed only weak seasonal patterns in case fatality and in the proportion hospitalized.

### Sensitivity analyses

The number of myocardial infarctions identified in the outpatient register without a record in the inpatient or cause of death data was small. Between 2019 and 2020, this number declined to an extent similar to the number of myocardial infarctions in our main analyses (11.2% compared to 10.3%).

## Discussion

The increased risk of myocardial infarction associated with Covid-19 infection, along with the ideas that monitoring of risk factors has been compromised during the pandemic, and that lockdowns have negatively influenced health behaviours, have led to widespread concern about increasing rates of heart disease following the global pandemic [16, 22–24, 30]. Our results do not support these concerns. Despite the high spread of COVID-19 across the Swedish population, we found that incidence rates of myocardial infarction continued to decline at least until the end of 2022, thus following the long-term downward trend observed already before 2020. While there is an indication that the declining trend may have halted among the oldest men, observed rates still lie well within predicted intervals. Even in Stockholm County, an area in which COVID-19 was already widespread during March and April 2020, when vaccinations were not yet available and medical staff was still inexperienced in treating the virus [31], we found no evidence for increasing rates of myocardial infarction.

Evaluating changes in the incidence of myocardial infarction is challenging, as rates are shaped by a complex interplay of long-term trends, seasonal fluctuations, and changes in the population at risk. Simple comparisons to earlier years can therefore lead to incorrect conclusions and to an overestimation of differences between the pandemic and prepandemic periods. We fitted expected rates for the years 2020 to 2022 based on the previous years’ trends and seasonal variation also considering changes in the composition of the population at risk. Even in these analyses, we found substantially lower incidence rates; approximately 900 fewer events occurred during the first pandemic wave than expected, a number corresponding to 13% fewer than expected myocardial infarctions during this period.

Competing risk of death from COVID-19 is one proposed mechanism behind the declining number of cardiovascular disease events. Severe COVID-19 infections and cardiovascular diseases share common risk factors [22, 30], and it is hence possible that the number of high-risk individuals depleted faster than the total population at risk, thereby not only reducing the total number of myocardial infarctions but also incidence rates. This hypothesis is, however, challenged by consistently lower incidence rates in areas outside of Stockholm County already in March and April 2020. These areas experienced virtually no deaths from COVID-19 in this early phase of the pandemic, yet introduced recommendations for older individuals to stay at home [31]. Furthermore, we analyzed changes in the composition of the population at risk with respect to age, sex, comorbidity, and care status and found no substantial changes during the pandemic.

The etiological mechanisms behind the notable decline in myocardial infarction in the early stage of a global pandemic are intriguing and remain to be studied further. Altered stress levels, lifestyle, and environmental factors, such as reduced air pollution during lockdown, may have contributed to lowering the risk of acute myocardial infarction [22]. While many of these factors operate through long-term accumulation of risk, factors that trigger myocardial infarctions in the short term, such as stress or air pollution, may contribute as well [32, 33]. Research has shown that air pollution can indeed affect the risk of myocardial infarction within weeks, days and even hours of exposure to pollutants [34–36]. Even despite the comparatively lenient restrictions during the pandemic, Swedish air pollution levels decreased substantially. WHO reported a roughly 30% lower mean annual concentration of NO_2_ fine particles and 18% lower concentrations of PM_10_ and PM_2.5_ particles during 2020 compared to 2018–2019 in Stockholm [37].

The absence of higher fatality and of higher proportions of patients dying before receiving care is noteworthy. Clinical processes and staff have been challenged during the pandemic; surgeries have been postponed, and waiting times for patients with many diseases have increased [38]. Indeed, delays in the care pathways of cardiovascular conditions as well as poorer treatment outcomes have been observed in some studies in low- and middle-income countries [16]. For the Swedish setting, the clinical register *Swedeheart* reported that the time to treatment of acute myocardial infarction had not been prolonged during the pandemic [26]. Reporting to this register is not mandatory and has declined during the pandemic [25, 26], but our study based on nationwide administrative data supports the conclusion that increased pressure on the Swedish health care system has not led to poorer outcomes for patients presenting with acute myocardial infarction.

Our study has several strengths. We use nationwide administrative data on the entire Swedish population, which allow us to derive precise estimates of person-time at risk and incident myocardial infarction. While reporting to clinical registers is prone to be disrupted once clinical processes are challenged and staff shortages occur, reporting to administrative registers is mandatory and has a high priority because it is directly linked to the reimbursement of health care costs. Sensitivity and positive predictive values for myocardial infarction in Swedish inpatient data have been shown to be excellent [28, 29], Specific ICD codes are available to encode a history of myocardial infarction, limiting the probability of misclassifying historical events as incident events. Nevertheless, we cannot rule out some misclassification. Our data did not allow us to identify myocardial infarctions for which patients did not seek any care, and it is further possible that causes of death are misclassified in some instances. However, this would only induce bias if misclassification changed systematically over time. Although one could argue that the accuracy of cause of death assignment has decreased under the pressure of the pandemic, medical scrutiny may have also been promoted by efforts to determine the presence of COVID-19 infection in deceased individuals. Either way, we obtained similar results when excluding data from death records, indicating that misclassification of cause of death cannot explain the pronounced declines in myocardial infarction incidence in Sweden. Finally, it should be noted that our study is limited to ages 60 and above but Swedish authorities reported that 14% of all myocardial infarctions occured in ages below 60 years as of 2022. Younger ages might have adopted different lifestyles than older people during the pandemic, and it is not certain that our findings can be generalized to the younger population.

## Conclusion

The incidence of myocardial infarction among individuals aged 60 + in Sweden continued to decrease between 2020 and 2022, despite concerns about an increased incidence of cardiovascular diseases during the COVID-19 pandemic. During the first wave of the pandemic, there was an additional decline in incidence rates. These declines were neither accompanied by increasing case fatality nor by lower shares of patients being hospitalized. Our findings support the conclusion that increased pressure on the Swedish health care system has not led to increased risks or poorer outcomes for patients presenting with acute myocardial infarction. Our work also suggests that the effect of COVID-19 on myocardial infarction risk is not substantial, as we found no increase in incidence at the population level, despite the large share of the population that has been exposed to COVID-19.

### Supplementary Information

Below is the link to the electronic supplementary material.

Supplementary file1 (DOCX 976 kb)

## Data Availability

Data were provided by the Swedish National Board of Health and Welfare and Statistics Sweden. Restrictions apply to the availability of these data, which are thus not publicly accessible. Pseudonymized data are, however, available from the authors upon reasonable request and with permission of the regional ethics board in Stockholm. Aggregated data on age-specific incidence rates as well as statistical code are available upon request from the corresponding author at anna.meyer@ki.se.
