# Supplementary material for: Continued decline in the incidence of myocardial infarction beyond the COVID-19 pandemic: a nationwide study of the Swedish population aged 60 and older during 2015–2022
Source: Eur J Epidemiol. 2024 Apr 23;39(6):605–12. doi: 10.1007/s10654-024-01118-4 (PMC11249421; doi:10.1007/s10654-024-01118-4)
Supplement: Supplementary file 1 — Supplementary file1 (DOCX 976 kb) [file 10654_2024_1118_MOESM1_ESM.docx]

**Supplementary materials**

**Continued Decline in the Incidence of Myocardial Infarction Beyond the COVID-19 Pandemic: A Nationwide Swedish Study 2015–2022**

Anna C. Meyer* (PhD)^1^, Marcus Ebeling (PhD)^1;3^, Enrique Acosta (PhD)^2;3^, Karin Modig (PhD)^1^

^1^ Unit of Epidemiology, Institute of Environmental Medicine, Karolinska Institutet, SE-17177 Stockholm, Sweden

^2^ Centre for Demographic Studies (CED), Barcelona, Spain.

^3^ Max Planck Institute for Demographic Research, Rostock, Germany.

**Corresponding Author:** Anna C. Meyer (anna.meyer@ki.se)


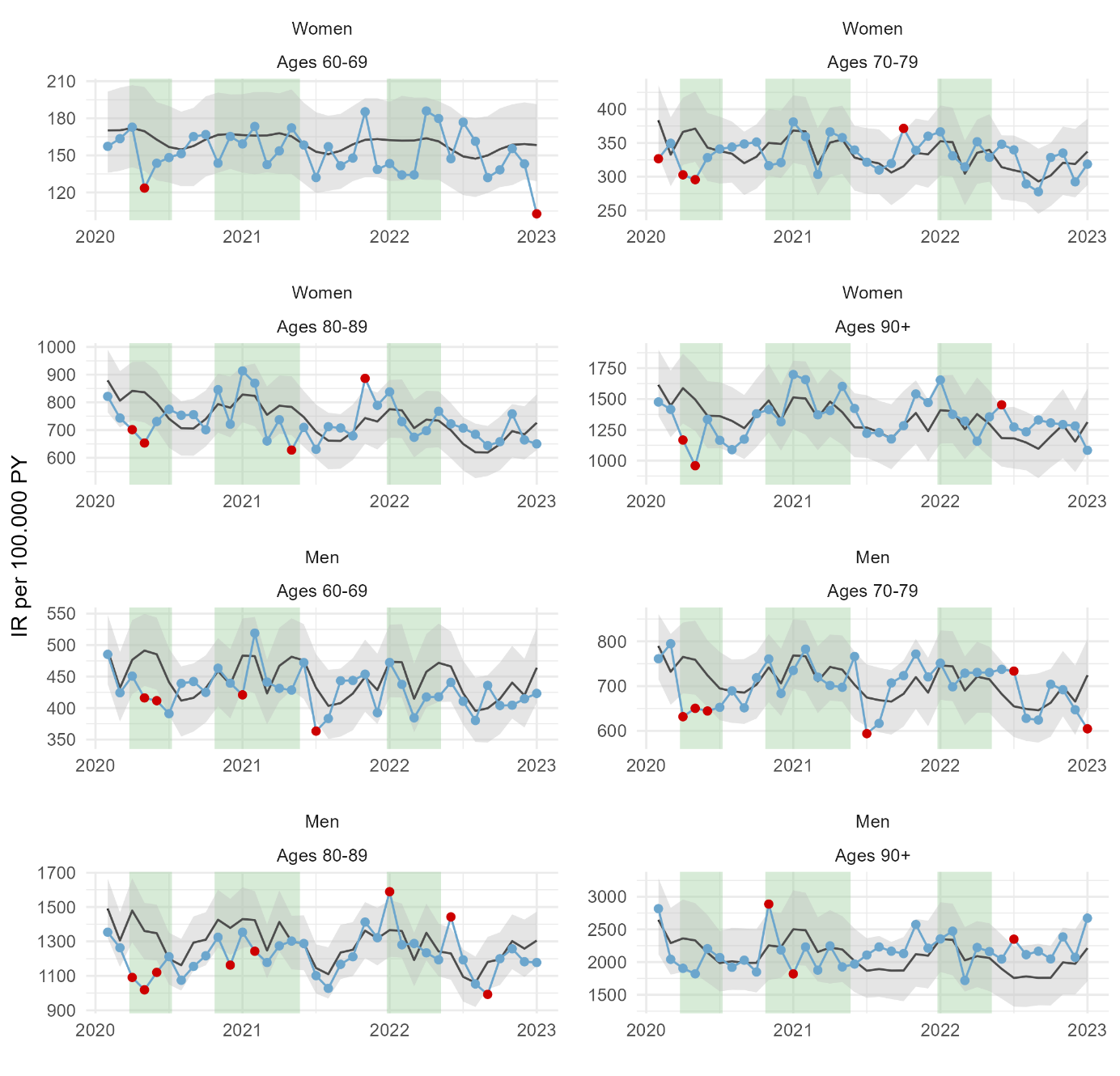


*Supplementary Figure 1:* *Expected and observed incidence rates of myocardial infarction per 100,000 person-years in the Swedish population over the age of 60 during March 2020 to December 2022, stratified by sex. Expected incidence based on trends since 2015 together with 95% prediction intervals shown in grey. Observations outside of predictions intervals are highlighted in red and the first three pandemic waves are displayed in green.*


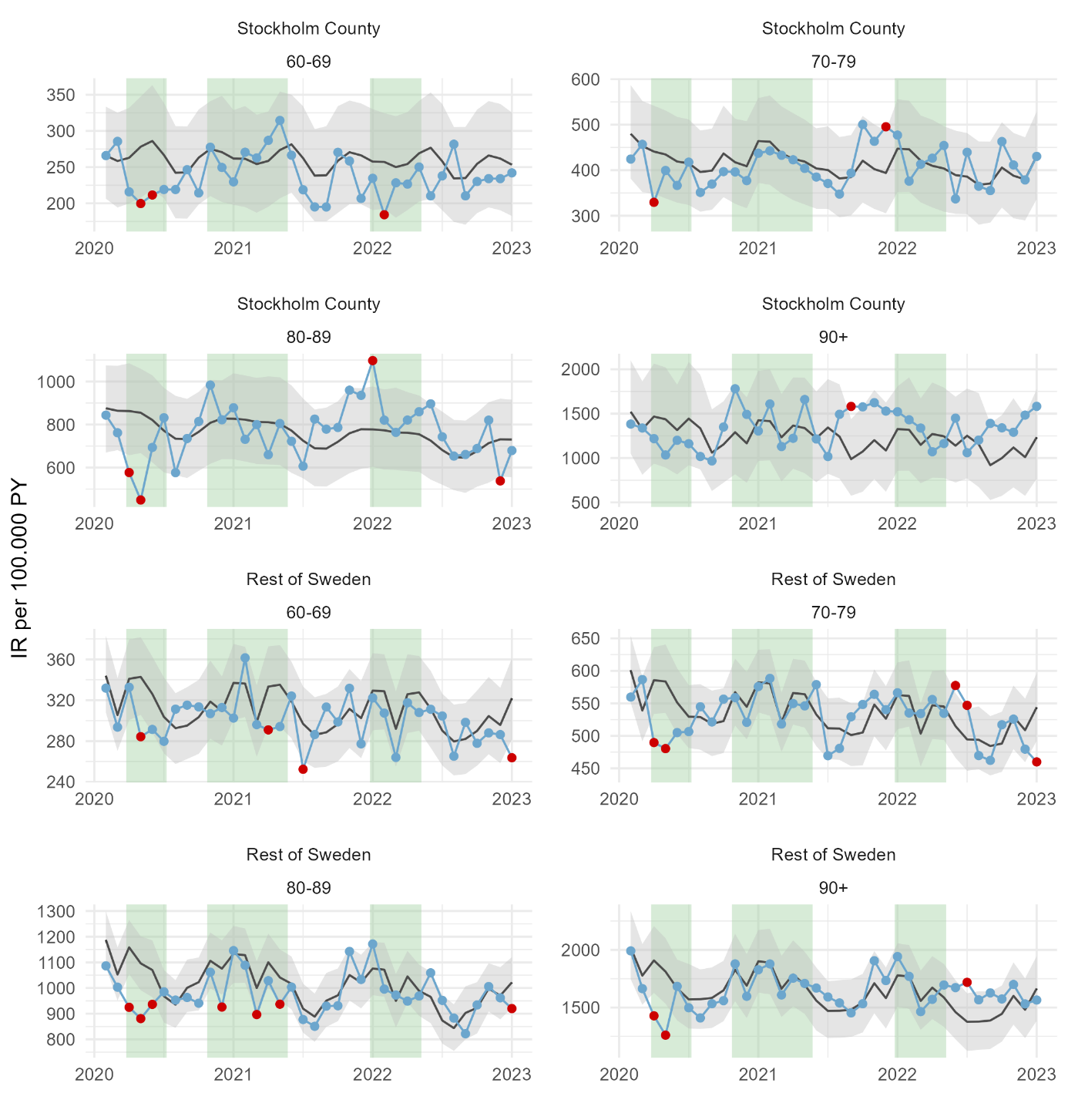


*Supplementary Figure 2:* *Expected and observed incidence rates of myocardial infarction per 100,000 person-years in Stockholm County and in the Rest of Sweden during March 2020 to December 2022, stratified by sex. Expected incidence based on trends since 2015 together with 95% prediction intervals shown in grey. Observations outside of predictions intervals are highlighted in red and the first three pandemic waves are displayed in green.*


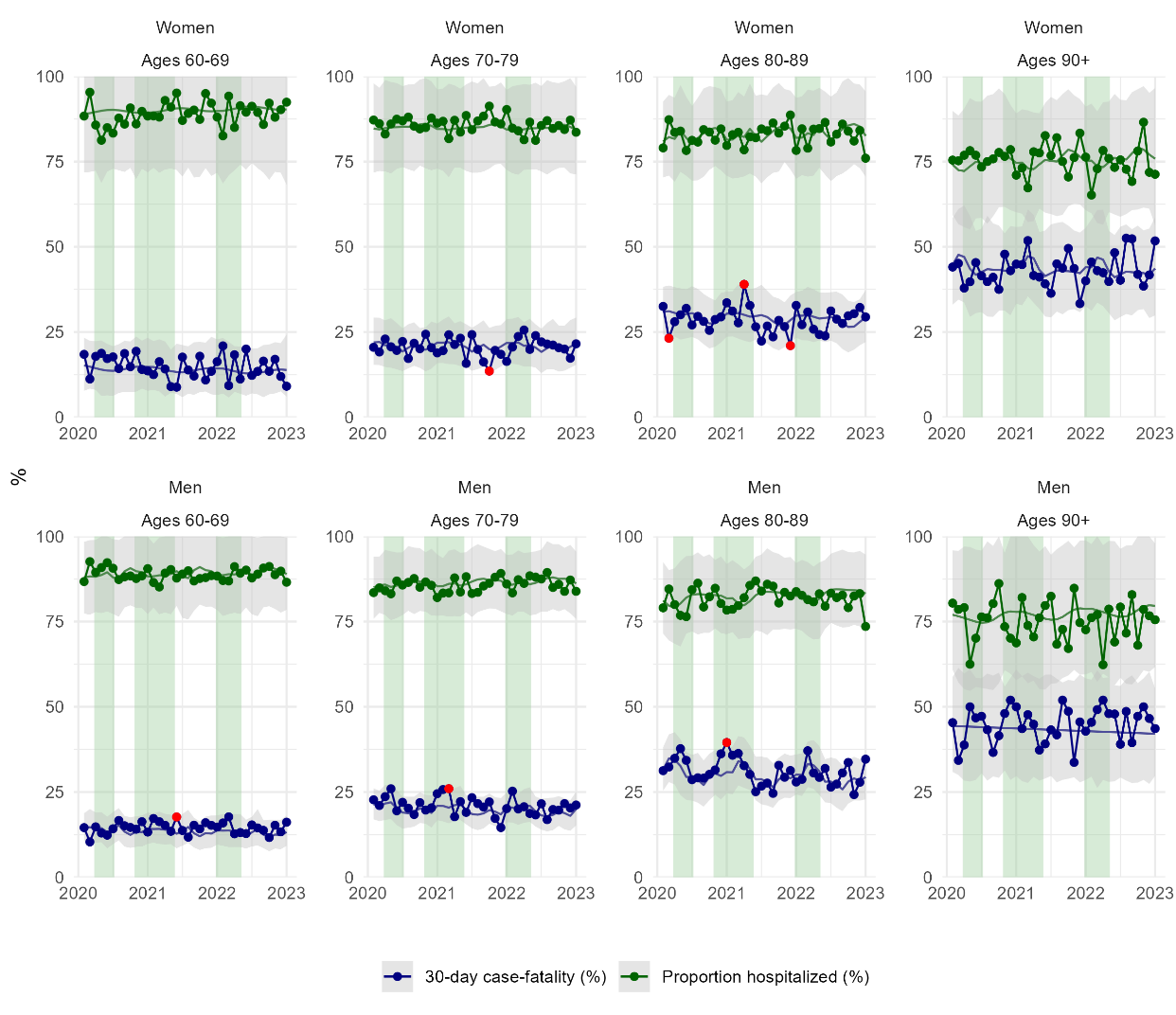


*Supplementary Figure 3: Proportion of myocardial infarction cases dying within 30 days (case fatality, blue graphs) and proportion of individuals with myocardial infarction receiving in hospital care (green graphs) in the Swedish population aged 60 stratified by age and sex, March 2020 to December 2022. Shaded areas show 95% prediction intervals. Observations outside of predictions intervals are highlighted in red and the first three pandemic waves are displayed in green.*
